# Accuracy of non-medical and medical individuals in identifying cerebral cortical abnormality from three-dimensional printed models of magnetic resonance images in children with hypoxic ischemic injury

**DOI:** 10.1007/s00247-023-05653-2

**Published:** 2023-04-11

**Authors:** Anith Chacko, Shyam Sunder B. Venkatakrishna, Sean Schoeman, Savvas Andronikou

**Affiliations:** 1 School of Clinical Sciences, Faculty of Medicine, University of Bristol, Bristol, BS28DX UK; 2 Radhiant Diagnostic Imaging SA Inc, Eastern Cape, East London, South Africa; 3 Department of Radiology, Children’s Hospital of Philadelphia, Philadelphia, PA USA; 4 Perelman School of Medicine, University of Pennsylvania, Philadelphia, PA USA

**Keywords:** Brain, Children, Hypoxic ischemic injury, Magnetic resonance imaging, Three-dimensional model, Three-dimensional printing

## Abstract

Effective communication of imaging findings in term hypoxic ischemic injury to family members, non-radiologist colleagues and members of the legal profession can be extremely challenging through text-based radiology reports. Utilization of three-dimensional (D) printed models, where the actual findings of the brain can be communicated via tactile perception, is a potential solution which has not yet been tested in practice. We aimed to determine the sensitivity and specificity of different groups, comprising trained radiologists, non-radiologist physicians and non-physicians, in the detection of gross disease of the cerebral cortex from 3-D printed brain models derived from magnetic resonance imaging (MRI) scans of children. Ten MRI scans in children of varying ages with either watershed pattern hypoxic ischemic injury (cortical injury) or basal-ganglia-thalamus hypoxic ischemic injury pattern with limited perirolandic cortical abnormalities and 2 normal MRI scans were post processed and 3-D printed. In total, 71 participants reviewed the 12 models and were required to indicate only the brain models that they felt were abnormal (with a moderate to high degree of degree of confidence). The 71 participants included in the study were 38 laypeople (54%), 17 radiographic technologists (24%), 6 nurses (8%), 5 general radiologists (7%), 4 non-radiologist physicians— 3 pediatricians and 1 neurologist (6%) and 1 emergency medical services staff (1%). The sensitivity and specificity for detecting the abnormal brains of the 71 participants were calculated. Radiologists showed the highest sensitivity (72%) and specificity (70%). Non-radiologist physicians had a sensitivity of 67.5% and a specificity of 75%. Nurses had a sensitivity of 70% and a specificity of 41.7%. Laypeople (non-medical trained) had a sensitivity of 56.1% and a specificity of 55.3%. Radiologists’ high sensitivity and specificity of 72% and 70%, respectively, validates the accuracy of the 3-D-printed models in reproducing abnormalities from MRI scans. The non-radiologist physicians also had a high sensitivity and specificity. Laypeople, without any prior training or guidance in looking at the models, had a sensitivity of 56.1% and a specificity of 55.3%. These results show the potential for use of the 3-D printed brains as an alternate form of communication for conveying the pathological findings of hypoxic ischemic injury of the brain to laypeople.

## Introduction

Hypoxic ischemic brain injury is a well-described spectrum of injury resulting from perinatal ischemia suffered by the neonatal brain. Magnetic resonance (MR) scanning is recognized as the method of choice to identify, characterize and establish the extent of injury [1]. Depending on the duration and severity of brain insult, brain MR scans in patients with hypoxic ischemic injury show characteristic features involving the cerebral cortex [1]. The broad patterns of injury fall into the following groups: (1) basal ganglia-thalamus pattern, (2) watershed predominant pattern of injury and (3) other patterns forming punctate white matter lesions and subcortical white matter involvement [1].

It is important to communicate the brain injuries and the regions affected not only to medical professionals involved in the children’s care but also to the patients, families and legal professionals representing them for compensation. Understanding the extent of hypoxic brain injury as it correlates to the radiology report is relevant to 3 distinct groups of people involved with the patient. The parents/family have a stake in understanding their child’s injury in a meaningful way so they can appreciate the course of development expected of their child. The non-radiologist clinicians involved in patient clinical care can better appreciate radiology reports substantiated with images/modeling [2]. Legal professionals (judges/lawyers) involved with determining and awarding appropriate compensation would benefit from less technical modeling of the brain that correlates with clinical severity of the condition. The use of standard multi-slice MR images (MRI) to meaningfully communicate the necessary information to the above groups in the above circumstances is difficult. Challenges arise because the involved parties do not possess the requisite medical knowledge needed to interpret diagnostic MRI. Interpretation requires detailed medical knowledge as it pertains to anatomy, spatial orientation of cross-sectional scans and pathology.

Evidence suggests that 2-dimensional (D) projections of MRI of hypoxic ischemic injury-associated pathology would be a useful adjunct to written reports. This has been achieved through “flat-earth” projections that represent the cortical surface of the brain in both Mercator (coronal reconstruction) and scroll (sagittal reconstruction) map forms [3, 4]. Both the Mercator and scroll projections have been used to visually represent pathology. The typical pattern of bilateral, symmetric injury seen in hypoxic ischemic injury has been demonstrated using these flattened brain surface images, as they provide an overview of the surface disease of the brain—*a bird’s-eye view*. One study demonstrated good sensitivity and specificity (> 60%) in a non-medically trained cohort, successfully identifying gross pathology in a brain with hypoxic ischemic injury [4]. The role of 3-D printed models can build upon the evidenced success of 2-D projections in the demonstration of watershed cortical volume loss in children with hypoxic ischemic injury [5]. The main benefit of using 3-D printed models is that the surface of the brain can be explored by touch, allowing the person viewing the injured brain to hold, rotate and touch injured portions of the brain in addition to having a bird’s-eye view. Scaled prints also allow for appreciation of the volume or size of the brain in relation to the hand holding it. It is assumed that the pertinent findings can be conveyed better than on 2-D MR images, especially to non-medical individuals. In this manuscript, we refer only to the surface cortical injury in the watershed, which has proven to be difficult to demonstrate according to S.A.’s experiences with more than 2,500 medicolegal cases in South Africa. The watershed is not well-described in the literature; therefore, this group has previously published 2 papers defining the watershed and the use of 3-D models to define the extent of the watershed [6, 7]. To the best of our knowledge, however, there is no current literature testing the understanding of radiologists or non-radiologists concerning the distribution of surface injury in hypoxic ischemic injury.

## Aim

To determine the sensitivity and specificity of different groups of individuals comprising non-medical laypeople and medical professionals (radiologists and non-radiologists) for the detection of abnormalities of the brain surface from 3-D printed models of children’s brains, created from diagnostic MRI studies.

## Materials and methods

Ethical approval for this work was obtained from the Faculty of Science Human Research Ethics Committee of the University of Bristol, England. We performed 3-D printing of 12 out of 40 segmented MRI scans randomly selected from a database of 243 patients. These included 10 children of varying ages who had suffered either partial prolonged hypoxic ischemic injury involving the watershed cortex at term or profound hypoxic ischemic injury at term involving the deep nuclei and with perirolandic cortical abnormalities. There were also 2 children selected with “normal” MRI scans according to the expert reports.

Creation of the 1:1 scale 3-D models involved several steps. First, DICOM (Digital Imaging and Communications in Medicine) of the 3-D T1‐weighted sequence data was converted into a NIfTI (Neuroimaging Informatics Technology Initiative) file. This was segmented by SPM12 (Statistical Parametric Mapping, The Wellcome Centre for Human Neuroimaging, UCL Queen Square Institute of Neurology, London, UK). Then, in MATLAB (MathWorks, Natick, MA), voxels were created which effectively assigned tissues to different classes according to signal intensity. The structure of the brain was then recreated using only solid tissue structures (grey and white matter, derived from the generated voxels). Here, the distinction between grey and white matter is less important than that between grey matter and cerebrospinal fluid. Then, the exterior surface was mapped in stereolithography STL (Standard Tessellation Language) format; internal volume data was discarded. The STL surface files, or “meshes,” are used in 3-D printing where the surface is deconstructed into a network of tetrahedral elements and printed in a layered format (slices). In the 3-D printing context, higher smoothness of a model reflects a lower detail print. The more STL data points, the more detailed the final print (referred to as “less smooth”) and the longer the printer processing time required. Printer failure is mitigated by repairing any meshing errors that occur when generating the STL file; repairs are made with the MATLAB applications MRIcros or isosurface. The fused deposition modeling (FDM) printing method, which is affordable, available and has been used for demonstrative purposes, was employed [2].

**Fig. 1:**
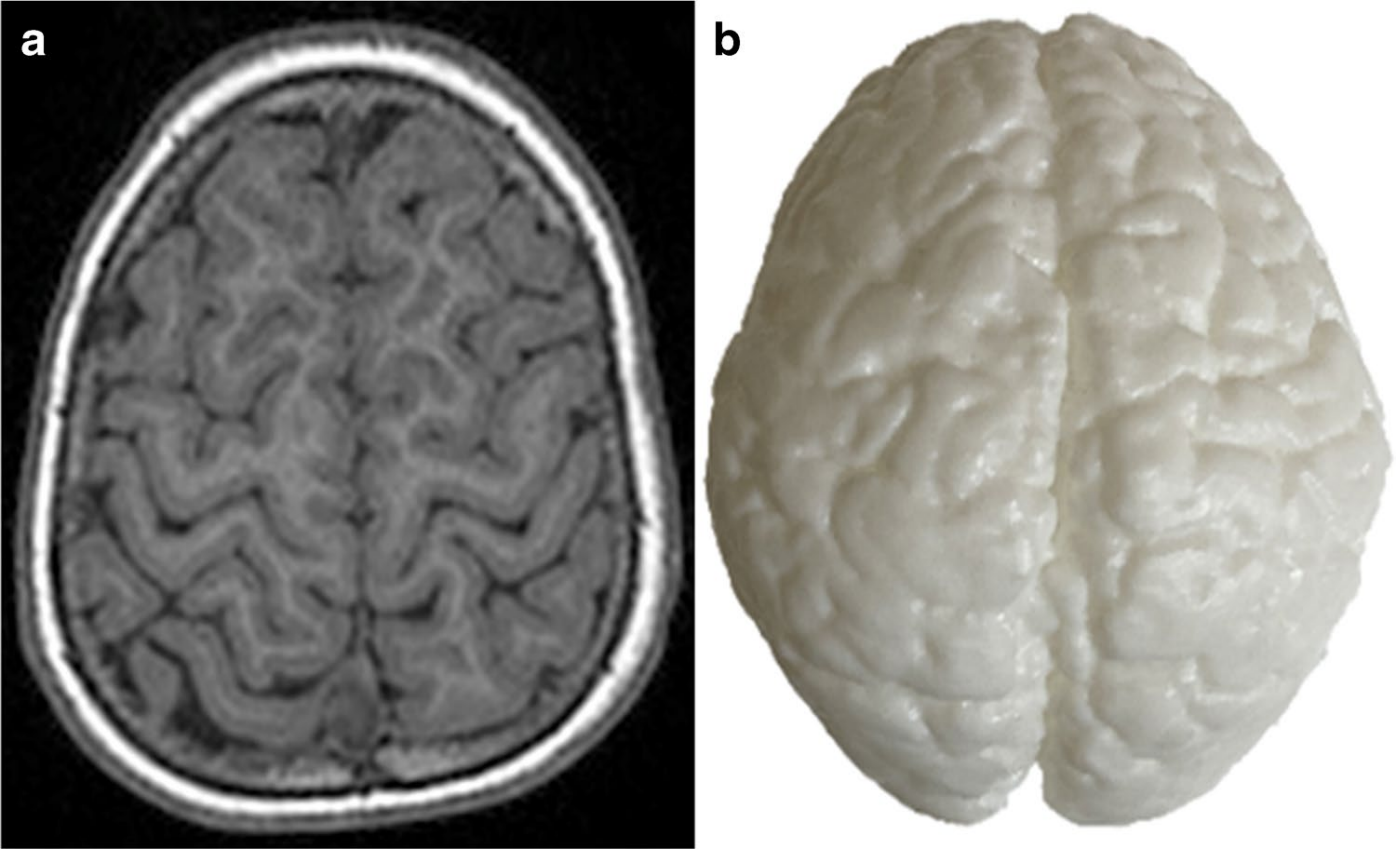
The normal cortex of the brain in a 5-year-8-month-old girl presenting with cerebral palsy. **a** Axial T1-weighted magnetic resonance image shows the cortical surface at the vertex. **b** A vertex view of the corresponding 3-dimensional printed model which accurately depicts the cortical surface

**Fig. 2:**
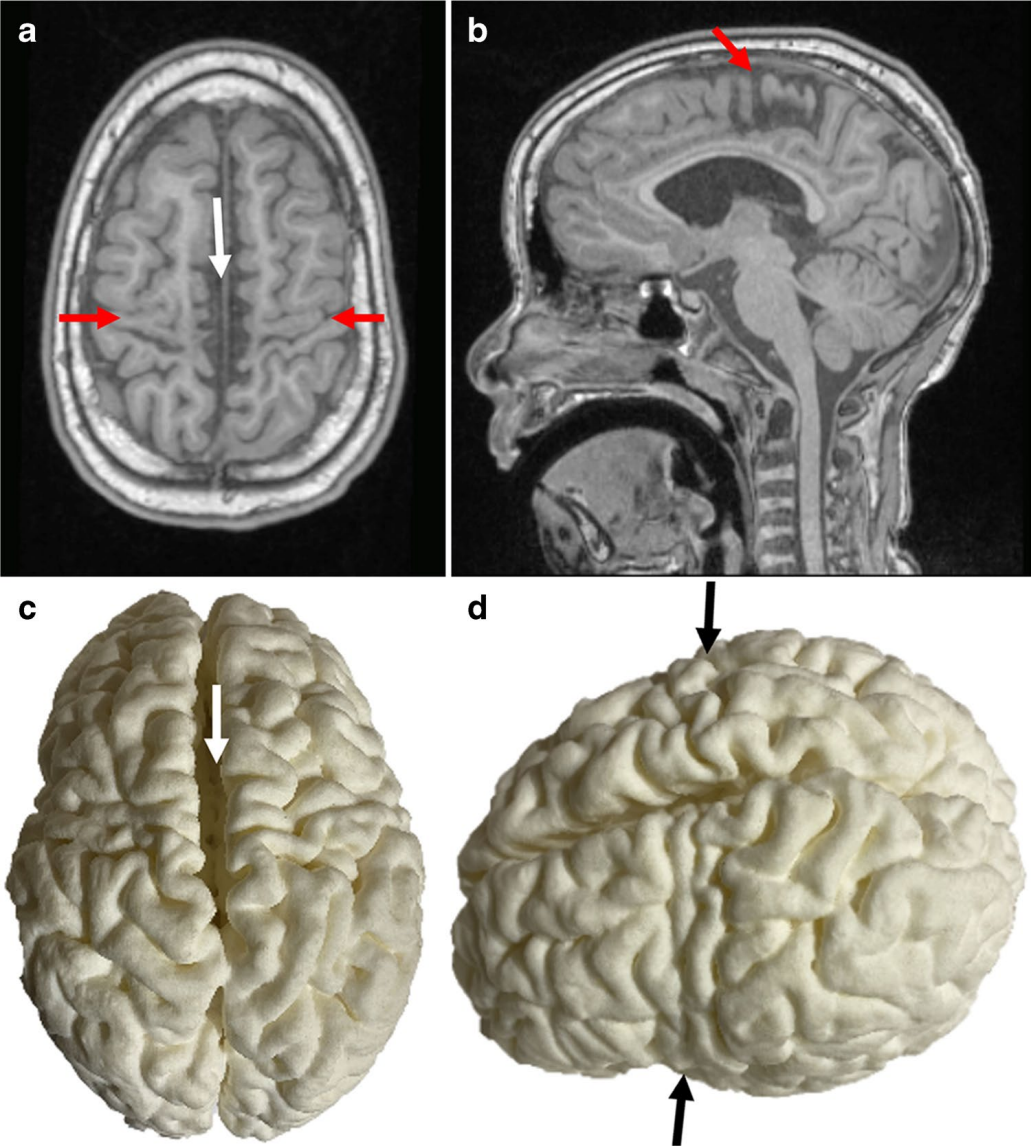
Images of an 11-year-6-month-old girl presenting with cerebral palsy. **a, b** Axial **(a)** and sagittal (**b**) T1-weighted magnetic resonance images show localized perirolandic atrophy and signal change (*red arrows*) and lentiform separation of the cerebral hemisphere (*white arrow*). **c** Vertex (**c**, **d**) and lateral oblique (**d**) 3-dimensional printed models with accurate depiction of the lentiform separation (*white arrow*) and perirolandic atrophy (*black arrows*)

**Fig. 3:**
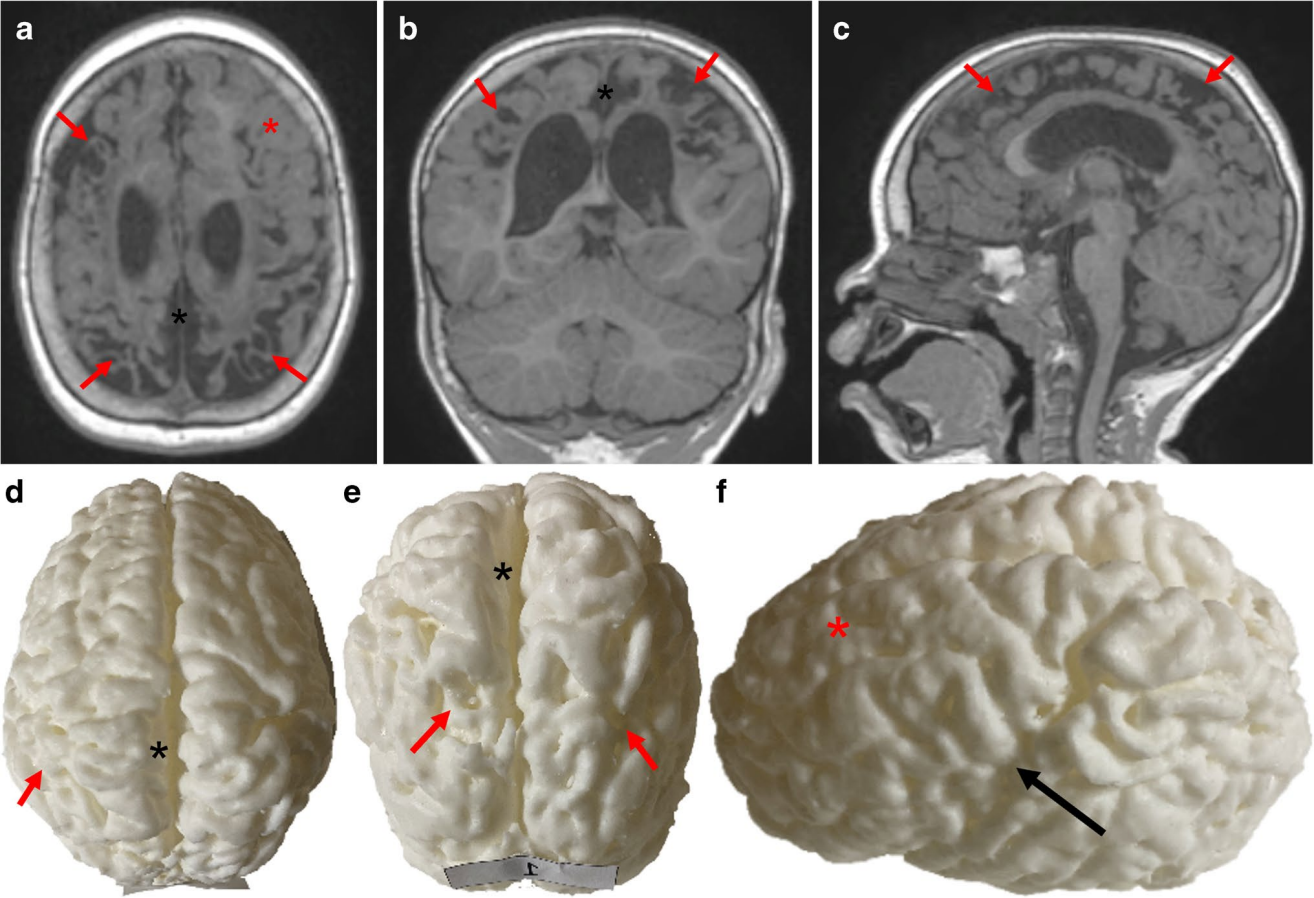
Images of a 2-year-2-month-old boy with cerebral palsy due to term partial prolonged hypoxic ischemia. **a–c** Axial **(a)**, coronal **(b)** and sagittal **(c)** T1-weighted magnetic resonance images of the brain and **(d–f)** vertex **(d)**, posterior **(e)** and left **(f)** side views of the corresponding 3-dimensional (D) printed model of the brain. Images show areas of injury with cortical atrophy (*red arrows*) clearly appreciated on the 3-D models, lentiform separation of the cerebral hemispheres (*black asterisks*) due to cerebral atrophy and volume loss in the parasagittal watershed regions, widening of the Sylvian fissure (*black arrow*) due cerebral atrophy and volume loss and relative sparing of the left anterior intervascular watershed (*red asterisk*)

The models were randomly placed onto a table and numbered from 1 to 12 for viewing by study participants, who were recruited from the local research and clinical environment of the principal investigator (A.C.), in South Africa. Representative MR images and the corresponding 3-D printed models of three cases are shown in Figs. 1, 2, and 3.

The participants were shown all 12 3-D printed models at the same time, laid out on a table. They were informed that the models were derived from both normal and abnormal MRI scans and were encouraged to handle the models while inspecting them. Participants were not provided with any interpretation training or any clinical information.

The participants were asked to use their own intuition or logic to indicate only the brain models which they thought, with a moderate to high degree of confidence, were abnormal. These were then recorded on a check sheet. These check sheets were collated with the expert MR report data and entered onto a spreadsheet—a score of 1 indicated that a 3-D model was selected as abnormal and a score of 0 was given if the case was not selected.

Sensitivity and specificity of the participants based on occupational group was then derived by averaging the true positives, true negatives, false positives and false negatives against the gold standard MRI interpretation, which uses standard 2-D images in evaluation and depiction or communication of hypoxic ischemic injury.

## Results

A total of 71 participants were involved in this study. The different individuals participating were classified as follows:

- Medical: There were 33 healthcare professionals (46%): 1 (1%) from emergency medical services, 6 (8%) nurses, 4 (6%) non-radiologist physicians (3 pediatricians and 1 neurologist), 17 (24%) radiographers and 5 (7%) general radiologists.
- Non-medical: There were 38 (54%) laypeople (cleaning, administrative, secretarial staff) with no prior medical training.

Sensitivity and specificity of the various groups is summarised in Table 1).

**Table 1:** Study group sensitivity and specificity

Study group	Sensitivity	Specificity
Radiologist	72.0%	70.0%
Non-radiologist doctor	67.5%	75.0%
Nurse	70.0%	41.7%
Radiographer	62.4%	55.9%
Lay person	56.1%	55.3%

## Discussion

The novel application of 3-D printing to medical fields has demonstrated a range of uses, such as for obtaining consent, in medical education and as evidence presented in court [8, 9]. A bilateral and symmetric pattern of brain injury is usually seen on MRI in hypoxic ischemic injury [10–13], and cortical injury is best demonstrated by techniques offering the viewer a bird’s-eye view of the brain, allowing appreciation of regional injury affecting both sides of the brain simultaneously.

The visualization of both sides of the brain surface through 2-D curved reconstruction of the surface of the brain from 3-D MRI acquisitions via scroll and Mercator map projections has been previously reported [3]. The benefits of using Mercator maps and their potential to communicate findings to laypeople so they can recognize bilateral, symmetric brain surface abnormalities identified on MRI have also been previously reported [4]. Andronikou et al. concluded that there was successful demonstration of cortical volume loss regionally as an alteration in the morphology on 3-D prints of children’s brain MR scans [2]. The current study has taken this further and tested laypeople’s ability to organically or intuitively (without any training) distinguish abnormal from normal brains by viewing and manually examining 3-D printed brains derived from children’s MRI scans.

This study demonstrates an association between laypeople and the ability to detect brain abnormalities without prior training/education. It was expected that the group with the highest sensitivity and specificity in identifying pathology would be the radiologists, followed by the non-radiologist medical doctors—this was confirmed and demonstrates that the methodology can be used to depict gross (macroscopic) pathology. Furthermore, results support the hypothesis that even without prior diagnostic training/exposure, there is an appreciable difference between normal and abnormal 3-D printed brains. The brain abnormality represents the macroscopic manifestations of the insult to the brain (i.e., hypoxic ischemic injury) that can be discerned by an untrained eye.

## Role of non-radiology trained personnel

There are different groups of individuals who may be involved personally or professionally with children who have suffered a hypoxic ischemic injury to the brain. Concerned parents must often navigate complex medical jargon in radiology reports which refer to even more complex diagnostic images of their children’s brains. This group of laypeople has a right to understand what has occurred and how it has affected their child’s brain. If the multitude of cross-sectional medical images is difficult to demonstrate to parents, then a single scale 3-D print of the child’s brain accurately derived from the medical diagnostic images may be able to bridge this gap in communication.

The other main group of people involved with these patients is those who care for them in hospitals and clinics. These include nurses, technicians working in radiology departments, primary care specialists, pediatricians and subspecialists (e.g., neurosurgeons and neurologists) who all have varying training which may or may not include exposure to medical imaging. In order to provide immediate care and plan future care, it is important for these group members to have a general understanding or accurate visual representation of the underlying pathology. The non-physician group may not have access to images, while the physician group may have access to images but a limited understanding of the findings. Identifying the degree of involvement and distribution of findings may play a significant role in planning care and for communicating the care to parents.

A third group of non-medical people that may need to understand the injury to the cortex in this specific patient group are the individuals involved in legal proceedings, such as lawyers and judges who handle matters related to compensation. Whether 3-D prints are used to demonstrate injured brain volume compared to normal brains or to demonstrate the particular bilateral symmetric distribution of cortical injury in hypoxic ischemic injury, the ability to hold up and view a 3-D printed and scaled brain in court will be an improvement compared to scrolling through thousands of cross-sectional diagnostic images. These traditional methods are often not to scale, and the images are not orientated in a way that is intuitive to laypeople. We did not test the ability of legal professionals to detect pathology on the 3-D printed models in this study, but this will form part of future work.

The lead author of this manuscript (A.C.) is an indigene of and lives and works in the Eastern Cape of South Africa—an underserved community and one suffering most from increased cases of cerebral palsy due to degradation of health services. The methodology used in the paper is based on open source (free) software, and we aim to allow other researchers or practitioners in other developing nations to relatively easily recreate the protocols and methods and thereby increase access to these techniques for demonstration of the cortical pathology of hypoxic ischemic injury. There were other options in terms of software and techniques that are more automated and said to be easier to use but are also significantly more expensive to acquire. This work as well as other publications stemming from the database are unique because the imaging is extremely delayed (as compared to the literature from developed nations which tends to examine the early phase of hypoxic ischaemic injury). Wheras imaging in the early phase with MRI uses DWI for detection of the insult and can pesudonormalize and miss pathology at approximately one week after the event, delayed imaging with routine MRI sequences shows permanent damage as well as any post-synaptic damage related to hypoxic ischemic injury.

## Limitations

The study team acknowledges limitations of this study. The retrospective nature of the study and the small number of participants who were radiologists or medical professionals (non-physician) are the two main limitations. A third limitation is that there were only 2 normal 3-D printed brains versus the 10 abnormal models. This affected the ability of the study team to analyze class differences. Finally, our results will have been affected by the range of disease included. In addition to cases with gross pathology, we also included 2 normal brains and 3 cases with subtle peri-Rolandic abnormality related to injury of the deep nuclei (not visible on the external surface). Our results may have been better had we included only cases with gross abnormality or worse with only cases of subtle abnormality. A larger study with a wider range of pathology is required.

## Conclusion

Our results show potential use for 3-D printed brains as an alternate form of communication for conveying the pathological findings of hypoxic ischemic injury to laypeople.

This paper demonstrates that 3-D printed brains can be appreciated as normal and abnormal by radiologists and physicians with adequate sensitivity. This shows potential for application to three interested groups involved with children who suffer from various degrees of hypoxic ischemic brain injury. However, non-radiologist medical personnel, parents/caregivers and legal professionals will require explanations including comparison with normal brains as they did not demonstrate adequate sensitivity intuitively.

Further investigation into the specific use for each of these groups needs to be conducted.

## Data Availability

The datasets generated during and/or analysed during the current study are available from the corresponding author on reasonable request.
